# Black Sheep: Pseudokinases Bucking the Trend to Reveal Non‐Catalytic Kinase Functions

**DOI:** 10.1002/iub.70125

**Published:** 2026-08-26

**Authors:** James M. Murphy, Natalia Jura, Peter D. Mace

**Affiliations:** ^1^ Walter and Eliza Hall Institute of Medical Research Parkville Victoria Australia; ^2^ Department of Medical Biology University of Melbourne Parkville Victoria Australia; ^3^ Drug Discovery Biology, Monash Institute of Pharmaceutical Sciences Monash University Parkville Victoria Australia; ^4^ Cardiovascular Research Institute University of California San Francisco San Francisco California USA; ^5^ Department of Cellular and Molecular Pharmacology University of California San Francisco San Francisco California USA; ^6^ Department of Biochemistry, Faculty of Biomedical Sciences University of Otago Dunedin New Zealand

**Keywords:** catalytically‐dead, protein interactions, pseudoenzyme, signal transduction

## Abstract

Pseudokinases are the catalytically‐dead counterparts of protein kinases and, over the past 20 years, have increasingly garnered attention as crucial signaling entities—comprehensively dispelling the possibility that they are merely evolutionary remnants or cellular passengers. The field has been framed by a sequence‐based definition of a pseudokinase, where the absence of one or more of the three critical catalytic residues required for phosphoryl transfer in conventional protein kinases has allowed their classification. As a result, pseudokinases have been defined by their dissimilarity to active kinases, meaning they are the outcasts or black sheep of the kinome. Pseudokinases are prevalent in nature, accounting for 10% or more of the kinase complement throughout phyla, and have been reported to mediate diverse functions in controlling the activities of other enzymes allosterically, mediating signaling complex assembly, serving as conformational switches and as negative regulators of signaling flux. Here, we review our current understanding of the varied pseudokinase functions as a window toward understanding non‐catalytic functions of conventional protein kinases, the challenges associated with defining pseudokinases—especially in cases where cryptic catalytic activities have been reported—and the emergence of pseudokinases as pharmacological targets.

## 1 Background

**FIGURE 1:**
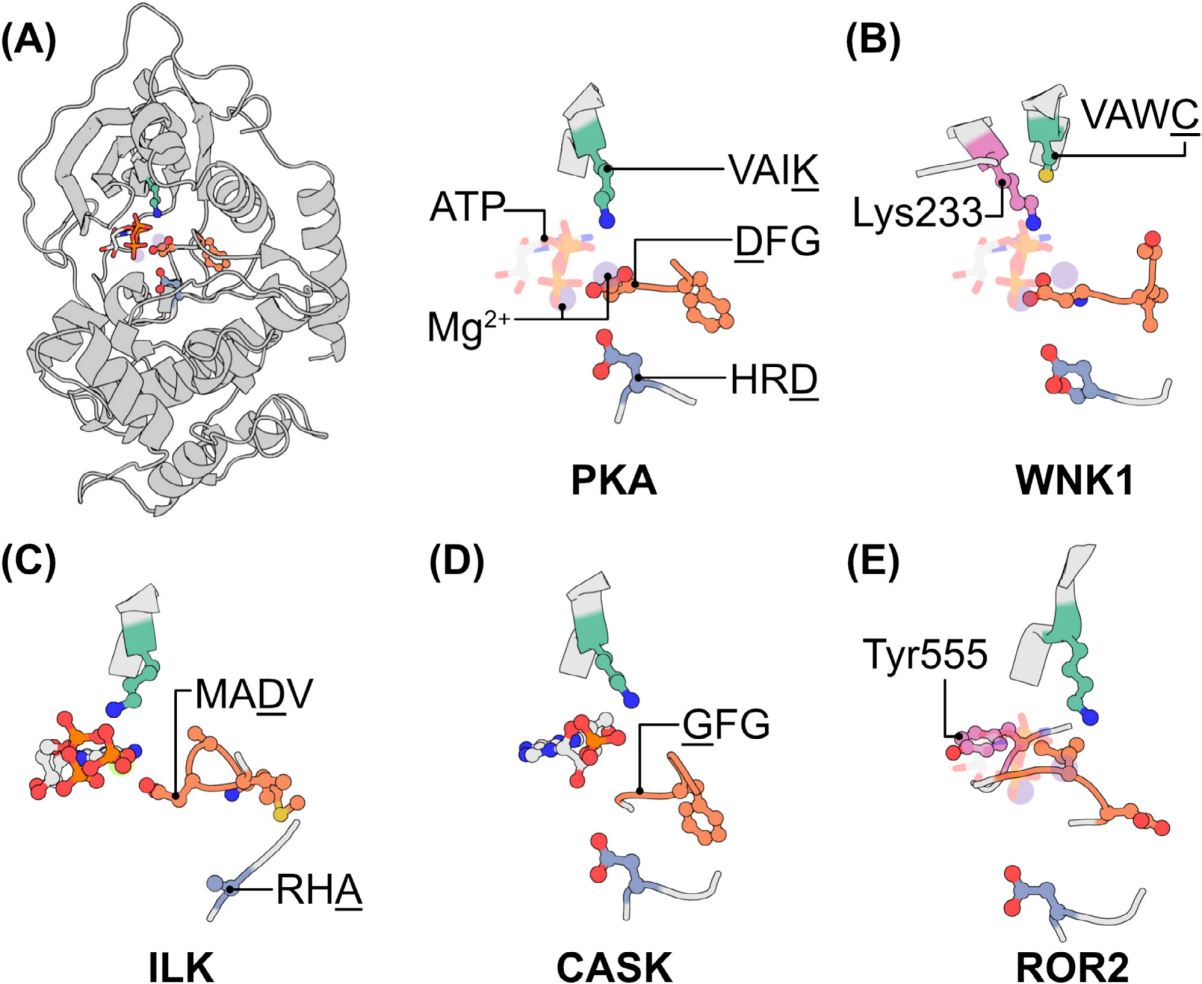
A comparison of a conventional and unconventional protein kinase active sites with pseudokinase “pseudoactive” sites. (A) The PKA active site in cartoon format, with closeup view of key catalytic features (PDB code: 1atp; [3]). (B–E) Examples of modified active sites illustrated in (B) WNK1 (PDB code: 4pwn; [4]), (C) ILK (PDB code: 3kmw; [5]), (D) CASK (PDB code: 3c0i; [6]), (E) ROR2 (PDB code: 3zzw).

The term “pseudokinase” entered scientific parlance in 2002, when Gerard Manning and colleagues noted that a substantial portion of the human kinome lacks features thought to be essential for catalysis [1]. However, the origins of pseudokinases are ancestral, with their presence across all phyla demonstrating a deep evolutionary history. Pseudokinases are defined by the absence of one or more of the three key catalytic residues previously identified as essential in catalytically‐active protein kinases [2]. These residues comprise: the VAIK lysine in the *β*3 strand within the N‐lobe of the kinase domain, which is essential for binding and orientating the *γ*‐phosphate of ATP for phosphoryl transfer; the HRD aspartic acid within the catalytic loop, which is the crucial catalytic residue for phosphoryl transfer; and the DFG aspartic acid at the start of the activation loop, which is critical for binding to the co‐catalytic Mg^2+^ ion (Figure 1A). The absence of one or more of these catalytic residues is known to compromise catalytic activity, and substitutions of each are routinely used to eliminate catalytic activity of conventional kinases to study their non‐catalytic functions (for example: [7, 8, 9]). However, there are some exceptions to the rule that we detail below. These examples illustrate the complexities of relying on a bioinformatic definition when evolution can co‐opt the kinase fold for noncanonical catalytic functions or evolve compensatory catalytic mechanisms for phosphoryl transfer. Importantly, pseudokinases are not to be confused with pseudogenes: pseudokinases are functional proteins expressed in all phyla, and serve diverse roles and critical cellular functions. Notwithstanding, pseudokinases are found in all kingdoms of life, and in eukaryotes represent ~10%–12% of protein kinases, in general. Some pseudokinase complement outliers include *S. cerevisiae* in which 3% of the kinome are pseudokinases; *Plasmodium falciparum* in which more than one third of the kinome are pseudokinases; and many plants species, which have elevated pseudokinase complements (12%–17%) contributing to innate immune responses [10]. Here we highlight the diversity of pseudokinase functions, and emergent thinking in the field, drawing principally on the mammalian examples that have driven our current mechanistic understanding.

Ancestrally, pseudokinases appear to have originated through duplication of conventional kinase gene loci, which lends itself to functional redundancy and the freedom for the duplicated gene to evolve distinct non‐catalytic functions within a signaling pathway [11]. In some instances, pseudokinases have no known ancestral ortholog of an active kinase, raising the possibility that pseudokinases might have emerged through lateral gene transfer events or their lineage has been lost in the fossil record [12]. Equally, where no ancestral active kinases are known in pseudokinase lineages, such as that of MLKL [13], one might speculate that, in some instances, active kinases could have evolved from pseudokinases. The origins of pseudokinases have been widely debated as a result of these many possibilities. While this has spurred some academic interest in restoring kinase activities by reintroducing crucial catalytic residues into pseudokinases [14, 15], ultimately the functions of pseudokinases do not depend on catalytic competency.

A pseudokinase was first described in the literature in 1988 when the lab of David Garbers identified a domain with kinase homology within the sea urchin guanylate cyclase protein [16]. At the time, the domain was designated the Kinase Homology Domain (KHD), which along with terms like kinase‐like domain and nonenzyme have subsequently arisen, complicating literature searches for new findings in the field. Fortunately, the emergence of the term pseudokinase in 2002 has yielded a clear definition and searchability, and has created a template for the emergence of a broader pseudoenzyme field in which catalytically‐dead analogs of most enzyme types are now known [17, 18, 19]. Here, we review the state of the field, including surprises and challenges arising from the bioinformatic designation of pseudokinases, and the insights into the non‐catalytic functions of kinases offered by detailed molecular study of pseudokinases.

## 2 When Is a Kinase Not a Kinase?

As discussed above, the formal definition of a pseudokinase is sequence‐based and revolves around deviations in the VAIK, HRD, and DFG motif sequences, whose compromise is known to diminish conventional kinase activities [1, 2, 20]. For this reason, pseudokinases might be considered the outcasts of the kinome, or the “black sheep,” united only by their dissimilarity to conventional protein kinases. This definitional framework has proven instrumental to building a pseudokinase field, but how does it accommodate situations in which the kinase domain fold has been repurposed by nature and evolves a noncanonical catalytic activity? Currently, prediction of noncanonical enzymatic functions remains enormously challenging and largely empirical. In cases where pseudokinases exhibit a noncanonical phosphoryl transfer activity [6, 21, 22], differentiating a trace catalytic activity from contaminating protein activities poses an enormous challenge, especially when such an activity is outside a physiologically relevant range. However, with a growing catalog of protein structures and biochemical characterization to serve as exemplars, and the ever‐increasing power of protein structure prediction, future assessment of noncanonical enzymatic functions may prove more computationally amenable.

To complement sequence‐based predictions, pseudokinases have been experimentally‐subclassified based on their ligand‐binding capacity into those that do not bind ATP or Mg^2+^ (Class 1), those that bind ATP but not Mg^2+^ (Class 2), those that bind Mg^2+^ or Mn^2+^ but not ATP (Class 3), and those that bind ATP and Mg^2+^ (Class 4) [23]. The capacity of a kinase domain fold to bind both ATP and Mg^2+^ is considered an essential criterion if a pseudokinase were equipped to catalyze phosphoryl transfer reactions. Because only ~25% of pseudokinases can bind both ATP and Mg^2+^, it appears that the vast majority of pseudokinases characterized to date lack the capability to catalyze phosphoryl transfer [23, 24, 25, 26, 27, 28, 29, 30]. Notwithstanding, simply binding ATP and Mg^2+^ need not assert a cryptic phosphoryl transfer activity, because the manner in which ATP is bound by a kinase or pseudokinase is crucial for catalytic competence, and this can only be revealed by experimental structures. The relief of evolutionary pressure to maintain classical kinase domain active site geometry among pseudokinases has lent itself to divergent nucleotide binding modes, as exemplified by FAM20A [31]. While not catalytically‐active itself, FAM20A is important to binding and activating the active paralog, FAM20C. Intriguingly, FAM20A has evolved an ATP‐binding function in which ATP binds in the reverse orientation to that typical of canonical kinases [31]. Evolution of a novel ATP‐binding mode is illustrative of the malleability of the kinase domain fold as a signaling scaffold and allosteric effector in cell signaling, and how kinase domains might evolve to function as metabolic sensors, such as through nucleotide sensing.

Currently, examples of noncanonical activities and residual kinase activity amongst pseudokinases are few, but incredibly interesting and illustrative of the versatility of the kinase domain fold. The WNK (with‐no‐lysine) kinases serve as a cautionary tale of reliance on bioinformatic classification. The canonical VAIK lysine is absent from the *β*3 strand in WNK kinases, and instead a compensatory lysine is present on the adjacent *β*2 strand, allowing some functional compensation and catalytic activity [32], as revealed from crystal structures of WNK orthologs [33] (Figure 1B). In other cases, such as JAK2 and HER3/ErbB3, the pseudokinase domains lack a conventional catalytic loop HRD motif and instead contain an HRN sequence, with variant sequences in other motifs synonymous with conventional kinase activity, such as DFG > DPG in the JAK2 activation loop and the absence of the Glutamate in the *α*C helix that pairs with the VAIK lysine, whilst retaining the capacity to bind both ATP and Mg^2+^ [23]. KSR2 has a variant ATP‐binding motif (VAIK > VAIR), but has retained HRD and DFG motifs, and binding to both ATP and Mg^2+^ [34]. As a result, under certain conditions, low levels of phosphoryl transfer activity have been reported [21, 22, 34], although whether this activity is of functional importance or merely reflective of the ATP‐binding function of the ancestral kinase fold remains unclear.

Equally, whether the ILK pseudokinase can catalyze phosphoryl transfer has been heavily debated [5, 35]. ILK has an even more divergent catalytic loop sequence (HRD > RHA) and can bind both ATP and Mg^2+^ [5, 23], but it is currently not clear from available protein structures how a noncanonical mechanism might facilitate catalysis (Figure 1C). One of the major challenges in deciphering whether ILK is active is that disrupting the counterparts of catalytic motifs within ILK compromises protein stability [36, 37]. Regardless, strategically‐designed mutations to ablate remaining plausible catalytic motifs in ILK do not compromise ILK signaling functions in biological assays [38], supporting the notion that cryptic activities—if indeed present—are secondary to the protein interaction functions [36]. Despite the structure of WNK1 being solved in 2004 revealing a compensatory N‐lobe lysine residue that equips the WNK kinases for catalytic activity [33], the first structure of a (dead) pseudokinase was that of CASK in 2008 [6], which was reported to possess a noncanonical catalytic activity despite the absence of the DFG motif (deviated to GFG; Figure 1D). Serial mutation of the remaining kinase catalytic motifs did not further diminish CASK phosphoryl transfer activity in the original report [6], suggesting that a contaminating protein is likely responsible for the observed activity. Accordingly, establishing whether a pseudokinase may have a residual catalytic activity remains an enormous challenge, especially when activities challenge limits of detection and bona fide substrates remain unknown.

Methods to define nucleotide‐binding potential, rather than catalytic activity, have proven essential to understanding pseudokinase functions [23, 24, 25, 26, 27, 28, 29, 30, 39]. These methods enable researchers to disentangle the potential to mediate catalysis from potential contaminants in recombinant protein preparations—in effect letting us examine the black sheep despite any interfering conventional ovines. The power of combining ATP‐binding potential with structural analysis to define catalytic competence is best exemplified by studies of ROR1 and ROR2 [23, 40]. While each possesses conventional VAIK, HRD, and DFG motifs synonymous with active kinases, each contains an aromatic residue within their hinge region that connects the N‐ and C‐lobes that, unusually for hinge aromatic residues, imposes a structural impediment to ATP binding (Figure 1E) [40]. These bulky hydrophobic residues occupy the ATP binding cleft and occlude ATP binding, rendering ROR1 and ROR2 catalytically dead despite harboring active site motifs resembling an active protein kinase [23, 40]. Thus, while some kinases are not defined as a pseudokinase based on sequence analyses because they retain the catalytic motifs typical of conventional protein kinases, a broader definition of pseudokinases should be considered to include catalytically‐dead kinases in which ATP binding has been lost.

## 3 The Hen's Teeth of the Black Sheep

**FIGURE 2:**
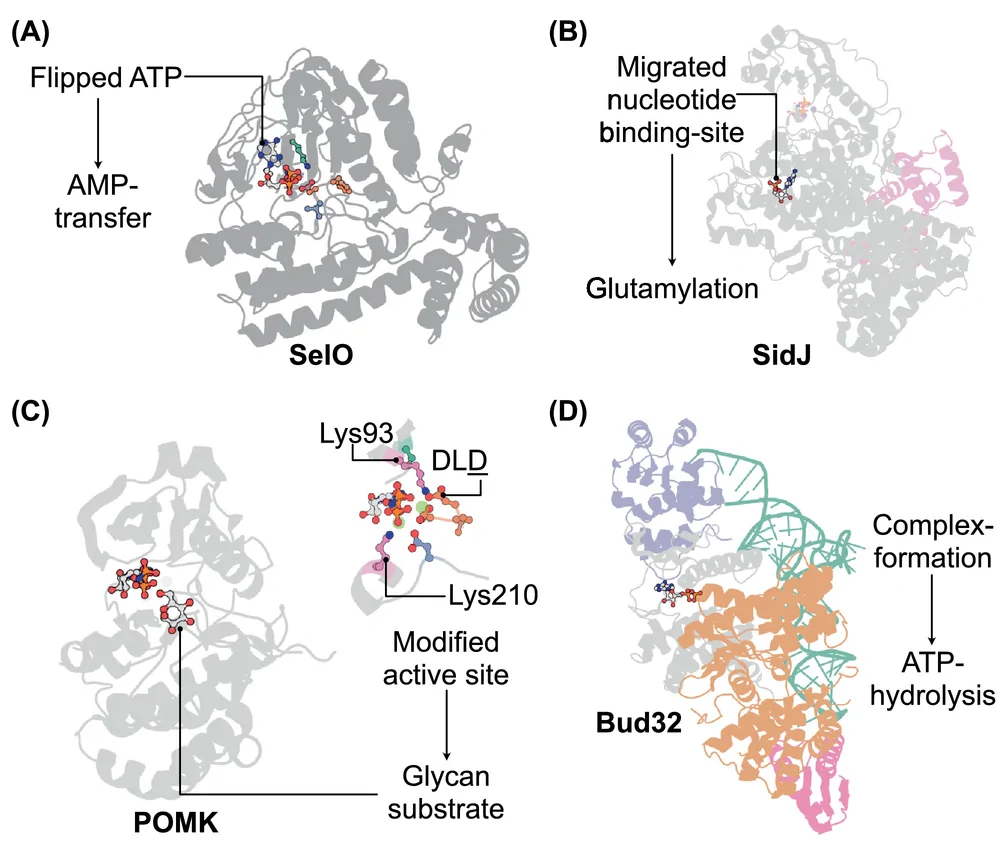
Alternative catalytic roles of pseudokinase domains. In all cases, pseudokinase domains are shown in grey. (A) Structure of SelO (PDB code: 6eac; [41]), illustrating a flipped ATP binding conformation to accommodate AMP rather than phosphate transfer. (B) Structure of SidJ with calmodulin bound (pink; PDB code: 6oqq; [42]), illustrating migration of the nucleotide binding site away from the conventional active site as it obtains glutamylation activity. (C) Structure of POMK (PDB code: 5gz9; [43]). A closeup of the modified POMK active site, which enables phosphorylation of glycan substrates, is shown. (D) Assembly of the Bud32:Cgi121:Kae1:Pcc1 tRNA complex (PDB code: 8up5; [44]), which enables ATP hydrolysis by Bud32.

Although rare cases of retention of residual kinase activities among pseudokinases have attracted much attention, the occasional instances of co‐option of the kinase fold by nature for the evolution of catalytic functions beyond phosphoryl transfer are fascinating. Here, we highlight four such proteins: SelO, SidJ, POMK, and Bud32 (Figure 2), each of which showcase the versatility of the kinase fold and highlight the challenges in categorically stating that pseudokinases lack activity based on catalytic motif divergence.

In the case of SelO, the ancestral catalytic loop HRD motif has diverged to HGV. In concert, the lysine of the ATP‐positioning VAIK motif in the N‐lobe has been retained, but with the neighboring sequence diverging markedly from those in conventional kinases. This divergence can be rationalized from the structure of SelO bound to ATP, which revealed that the SelO kinase domain fold has evolved to accommodate ATP in a reversed orientation compared to conventional protein kinases [41]. This observation proved enlightening and led to SelO being biochemically established as an AMPylating enzyme where the kinase fold has evolved to transfer AMP, rather than the *γ*‐phosphate, to serine, threonine, and tyrosine residues in substrate proteins [41].

Using similar approaches, the Tagliabracci lab identified the *Legionella* protein, SidJ, as containing a kinase domain fold in which the ATP‐binding site has been highly renovated through evolution [42, 45]. Owing to deviations within the catalytic loop sequence, the ATP‐binding site has migrated from the canonical position in a protein kinase, leading to the nucleotide nestling closer to the C‐lobe (Figure 2B). Concordantly, these renovations in the active site have facilitated a distinct catalytic function in SidJ: Calmodulin‐ and ATP/Mg^2+^‐dependent polyglutamylation of neighboring proteins encoded within the same genomic locus. Binding of host cell Calmodulin equips SidJ to bind ATP/Mg^2+^, which enables formation of an adenylylated intermediate and subsequent glutamylation of SidE—a critical step in attenuating the E3 Ubiquitin ligase activity of SidE, which would otherwise compromise host cell viability.

Protein O‐Mannosylation Kinase (POMK; SgK196) presents an even more intriguing case of acquired catalysis. Compared to canonical protein kinases, the ATP‐binding VAIK motif is lost and the two remaining critical catalytic motifs have highly divergent sequences, leading to categorization as a dead kinase. However, through compensatory substitutions, POMK has evolved kinase activity toward carbohydrate substrates, such as *α*‐Dystroglycan, which is an essential step in a sophisticated process of glycan chain elongation necessary for *α*‐Dystroglycan function in basement membranes [46, 47]. Like in WNK kinases, the VAIK motif is absent and the ATP‐positioning lysine instead migrated to the adjacent *β*2 strand (Figure 2C). Unlike in conventional protein kinases, this lysine does not form an ion pair with a Glu in the *α*C helix, but instead with an Asp that occurs in place of the canonical kinase DFG glycine within a DLD sequence [47]. Unusually, the catalytic loop forms a short helix that contains a crucial ATP‐binding lysine (K210 in human POMK), preceded by an MCD sequence, which occurs in place of the conventional kinase catalytic motif, HRD. This Asp residue serves as the catalytic residue, as in conventional kinases and, unusually, the adjacent Cys (C204 in human POMK) forms a disulfide with C244 in the activation loop, which augments active site structure and serves a critical role in substrate recognition [47].

Another intriguing example of a pseudokinase is the ancestral KEOPS (Kinase, Endopeptidase and Other Proteins of Small size) complex component, Bud32. While Bud32 contains the key catalytic residues typical of a conventional kinase, albeit within variant sequences (VAIK > AVIK; HRD > HGD; DFG; human Bud32/PRPK sequences), Bud32 is catalytically‐dead when prepared recombinantly [44, 48]. Intriguingly, Bud32 can hydrolyze ATP when part of complex with Cgi1, Kae1, and a substrate tRNA, where Kae1 projects a lysine into the Bud32 active site to facilitate ATPase activity [48], although Bud32 does not transfer phosphate to a protein substrate, only to water (Figure 2D). Such a mechanism is analogous to GTPase molecular switching, where hydrolysis of GTP to GDP is enabled by a catalytic residue projected into the active site by an auxiliary GTPase activating protein (GAP). Bud32 ATPase activity, and thus the Kae1 lysine, are pivotal to licensing Kae1 to carry out the ancestrally‐conserved and essential N^6^‐threonylcarbamoyl adenosine modification (t^6^A) in tRNAs. Collectively, these examples showcase the versatility of the kinase domain scaffold and the critical importance of protein structure to determining whether pseudokinases predicted to lack kinase activity have instead evolved a distinct catalytic function.

## 4 If Not Active, Then What?

**FIGURE 3:**
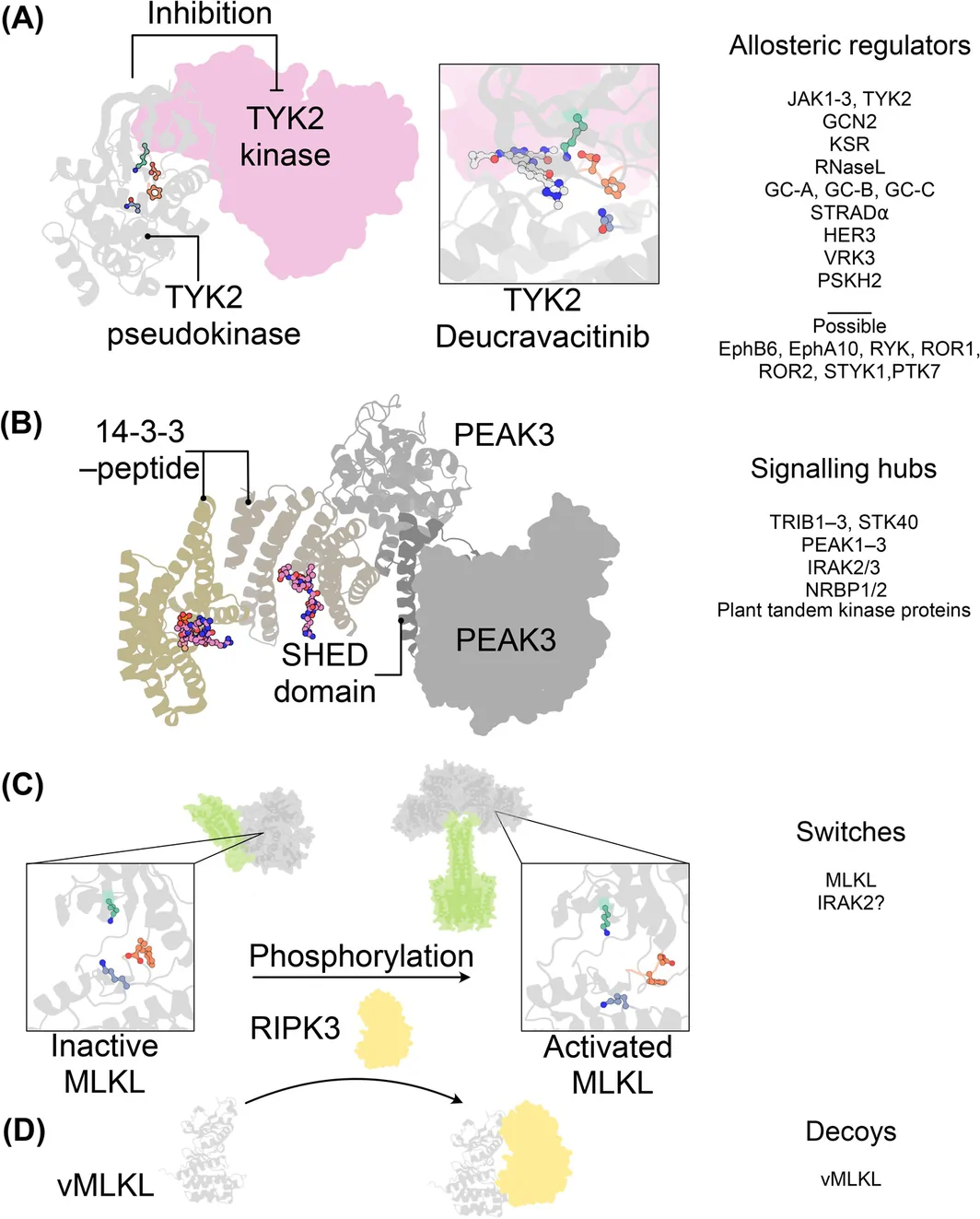
Exemplar modes of pseudokinase action. One example is illustrated for each, with further examples tabulated in the right‐hand column. (A) Allosteric regulation of enzymatic activities by pseudokinases. Allosteric regulation of TYK2 kinase activity by interactions between the pseudokinase and kinase domains (PDB code: 4oli; [51]). Inset is the structure of TYK2 active site with deucravacitinib bound (PDB code: 6nzp; [52]). Other examples of possible functions for pseudokinases in allosteric regulation tabulated include EphB6, EphA10, ROR1, ROR2, STYK1 and PTK7 [27, 40, 53, 54]. (B) Nucleation of signaling hub assembly. The PEAK3 14–3‐3 complex, illustrating PEAK3 oligomerization via the SHED domain, and further assembly of a signaling complex with 14‐3‐3 domains, in this case bound to an N‐terminal peptide from PEAK3 itself (PDB code: 8dp5; [55]). Other examples of pseudokinases that nucleate signaling complex assembly include other PEAK family members [55, 56, 57, 58, 59, 60], the TRIB family [25, 61, 62, 63], IRAK2 and IRAK3 [26, 64, 65, 66, 67], NRBP1 and NRBP2 [68], and plant tandem kinase‐pseudokinase pairs [69]. (C) Pseudokinases can serve as molecular switches. MLKL transitions between a monomeric, inhibited form to an active oligomeric pore‐forming conformation upon phosphorylation of the MLKL pseudokinase domain activation loop by RIPK3 [29, 30, 70, 71]. MLKL activation by RIPK3 is accompanied by exposure of the executioner four‐helix bundle domains and elongation the “brace” helices that connect the killer four‐helix bundle (both green) and regulatory pseudokinase domains. Inset active site structures based on crystal structures from PDB codes 7jw7 and 7jxu [71], for inactive and active conformations, respectively. (D) Pseudokinases acting as competitors in signaling pathways. Viral MLKL acts as a decoy to sequester RIPK3 and prevent cellular MLKL activation [72]. Surface silhouettes of vMLKL are based on Alphafold3 models.

The vast majority of proteins bioinformatically classified as pseudokinases lack catalytic activities, which raises the question of why they have been retained by nature throughout evolution if not to perform a catalytic reaction. Detailed structure–function studies of pseudokinases have proven instrumental in cataloging four principal functions, each of which revolves around pseudokinases serving as protein interaction domains [49, 50]. These comprise: (a) serving as allosteric regulators that promote or negate the activities of bona fide kinases or other enzymes; (b) nucleating the assembly of signaling complexes; (c) acting as molecular switches in signaling pathways; and (d) acting as substrate sinks or attenuating assembly of active holoenzymes to thwart signaling (Figure 3). These functions align with the generally characterized roles of pseudoenzymes more broadly [18, 49, 50], with pseudokinases providing quintessential examples of most classes of pseudoenzyme function. The catalytic functions of conventional protein kinases, especially identification of their substrates, have been a historic focal point, meaning their non‐catalytic functions have been often overlooked. As a result, the identification of the aforementioned four classes of pseudokinase action has provided an important window into the potential of conventional, catalytically‐active protein kinases to perform non‐catalytic functions. Here, we exemplify how these principles might apply to catalytically‐active kinases, with reference to the above‐mentioned classes of pseudokinase function. (a) Catalytically‐active HER/ErbB [73] and RAF [74] family members have been shown to act as allosteric activators of other members of their respective families. (b) Innate immune kinases, such as IRAK1/IRAK4 [75] and RIPK1 [76], have been reported to nucleate assembly of signalosomes in Toll‐like receptor and necroptotic signaling, respectively. (c) Kinase domain activation loop phosphorylation has been shown widely to induce an inactive‐to‐active conformation [77], often exemplified by the insulin receptor intracellular domain [78]. Lastly, (d) RIPK3 kinase binds and restrains the killer pseudokinase, MLKL, in a dormant form prior to receipt of upstream cues that provoke MLKL recruitment to the necrosome and activation via RIPK3‐mediated phosphorylation [70, 71, 79].

### 4.1 Pseudokinase Regulation of Conventional Enzymes

The origins of many pseudokinase domains from gene duplication events are best illustrated in cases where pseudokinase domains occur in tandem with conventional kinase domains within the same protein. In GCN2 and the Janus kinases (JAKs)—JAK1‐3 and TYK2—a pseudokinase domain occurs adjacent to a conventional kinase domain and has speciated to perform regulatory functions of the kinase domain's activity. In the case of the JAKs, the pseudokinase domain binds to the adjacent tyrosine kinase domain and suppresses catalytic activity [51, 80, 81] (Figure 3A). This inhibition can be overcome by upstream phosphorylation events, as well as by trans‐phosphorylation enabled by juxtaposition of JAKs upon ligand binding, dimerization and reorientation of the cytokine receptors to which they are associated [82]. The pseudokinase domain of GCN2 appears to serve a distinct function by facilitating GCN2 dimerization: a crucial step in trans‐phosphorylation and activation of the adjacent serine/threonine kinase domain and licensing GCN2 to participate in stress signaling [83]. Beyond occurring in tandem with conventional kinase domains, pseudokinases are also observed adjacent to other catalytic domains, such as guanylate cyclases. Indeed, the first reported example of a pseudokinase domain in nature was observed within a gene encoding a sea urchin guanylate cyclase [16]. Now, our appreciation has grown for the importance of the pseudokinase domain in regulating the activity of the adjacent guanylate cyclase domain through detailed biochemical studies of GC‐A, GC‐B, and GC‐C [84, 85]. These studies indicate that ATP binding to the pseudokinase domain is critical for domain rigidity and engagement of the adjacent guanylate cyclase domain to promote the latter's catalytic activity. Analogously, the binding of the 2′,5′‐oligoadenylate (2‐5A) second messenger to a cleft between the N‐terminal ankyrin repeat domain and pseudokinase domain reconfigures the conformation of the adjacent nuclease domain within RNaseL to induce its nuclease activity [86]. This mechanism illustrates the versatility of pseudokinase domains as sensors of second messengers and metabolites, beyond nucleotides like ATP. The pseudokinase conformation stabilized by ATP or second messenger binding encodes crucial information that is transmitted to adjacent domains to induce or suppress their catalytic activity.

Beyond regulation of adjacent enzymatic domains, pseudokinases are known to bind and regulate the activities of conventional kinases and other enzymes in trans. For example, activity of the tumor suppressor kinase, LKB1, is known to rely on formation of a ternary complex with a scaffold, Mo25*α*, and the pseudokinase, STRAD*α* [87, 88]. ATP binding appears to be a critical factor in imposing a closed, active‐like conformation of STRAD*α* that is capable of engaging Mo25*α* as well as binding to, and activation of, the LKB1 kinase domain. Notably, substitutions in STRAD*α* that disrupt pseudokinase domain integrity are causative of polyhydramnios, megalencephaly, symptomatic epilepsy (PMSE) syndrome [87, 88], underscoring the importance of ATP binding and domain stability in facilitating LKB1 catalytic activity.

Detailed structural studies have informed our understanding of how another pseudokinase, HER3/ErbB3, can allosterically activate catalytically‐active HER receptors *in trans* [89]. HER3 is a transmembrane receptor tyrosine pseudokinase, in which the C‐terminal region is intracellular and contains a pseudokinase domain. The HER3 pseudokinase domain can bind head‐to‐tail to other active HER receptor kinase domains upon growth factor‐induced receptor dimerization, and allosterically promote EGFR or HER2 catalytic activity and downstream signaling, mirroring the mechanism by which EGFR kinases transactivate within the EGFR homodimer. Whether similar allosteric functions in crosstalk with catalytically‐active transmembrane tyrosine kinase receptors are exerted by the other transmembrane tyrosine pseudokinase receptors—EphB6, EphB10, RYK, ROR1, ROR2, STYK1, and PTK7—remains to be established, as reviewed in detail recently [53].

In addition to regulation of conventional kinases, pseudokinase domains can also regulate activities of other enzymes. For example, the pseudokinase, VRK3, was reported to bind and activate the catalytic activity of the phosphatase, VHR/DUSP3, which in turn dephosphorylates ERK to selectively diminish its catalytic activity in the nucleus [24, 90]. With the recent acceleration of interactor discovery enabled by mass spectrometry‐based proteomics, we anticipate we are on the cusp of vastly expanded knowledge of how pseudokinases might bind and regulate the activities of kinases and other enzyme types to modulate signaling flux, as recently demonstrated for the PSKH2 pseudokinase [15].

### 4.2 Pseudokinases as Signaling Hubs

Another prevalent function of pseudokinases is to nucleate the assembly of large signaling complexes. Such complexes include bringing together bona fide enzymes and their substrates through multivalent interactions, as demonstrated for the Tribbles family pseudokinases. Structural and biochemical studies of TRIB1, TRIB2, and STK40 have revealed the importance of the pseudokinase domain for engagement of C/EBP*α* and a unique C‐terminal extension that recruits the E3 ubiquitin ligase, COP1 [25, 61, 62, 63]. Assembly of the COP1‐TRIB‐EBP*α* holocomplex leads to ubiquitylation of C/EBP*α* to induce its proteolytic turnover, which can drive oncogenic signaling synonymous with acute myeloid leukemia [91, 92, 93].

In addition to assembling enzyme‐substrate complexes, pseudokinases can also localize large signaling complexes within the cell. For example, the PEAK pseudokinases (PEAK1/SgK269, PEAK2/SgK223, PEAK3) contain an oligomerization domain termed the SHED domain adjacent to the pseudokinase domain, which promotes their dimerization and possible heteromerization, aided by binding to 14–3‐3 scaffolds [55, 56, 57, 58, 59, 60] (Figure 3B). The extensive N‐terminal domains of PEAK1 and PEAK2, and substantively shorter N‐terminal region of PEAK3, provide a platform to recruit additional signaling effectors through multivalent sites, such as via SH3 domain recognition and binding to phospho‐recognition domains, including SH2 domain‐containing proteins. Together, PEAK proteins have the capacity to assemble large multiprotein signaling complexes, a property that is likely beyond their ability to redirect growth factor‐induced signaling, for example from mitogenic to migratory outcomes in the case of PEAK1 [94].

Another recent illustration of nucleation of large signaling complexes mediated by pseudokinases is the assembly of a WNK1 signalosome by the pseudokinases, NRBP1 or NRBP2, which may also be considered WNK5 or WNK6, respectively, owing to their sequence similarity to WNK kinases [68]. NRBP1 and NRBP2 lack each of the core kinase catalytic motifs and possess a divergent Gly‐rich loop sequence, and NRBP1 is known to not bind ATP [23], consistent with a function principally as a protein interaction module. Precisely whether NRBP1 and NRBP2 serve to allosterically regulate the activity of WNK kinases within a supercomplex, or to nucleate supercomplex assembly, or whether both possibilities can co‐occur raises an interesting prospect that, in some cases, pseudokinases may confer a blend of functions in enzyme allosteric regulation, nucleation of signaling complex assembly, and/or serving as molecular switches.

Although knowledge is still emerging, the IRAK2 and IRAK3 pseudokinases have been implicated as signaling hubs in Toll‐like receptor (TLR) and IL‐1 receptor signaling [26, 64, 65, 66, 67]. Each of these pseudokinases appears to play a context‐specific role within supramolecular assemblies termed the Myddosome, in which the adaptor protein, MyD88, forms large filaments and recruits IRAK2 and IRAK3 pseudokinases, and the conventional kinases, IRAK1 and IRAK4, via their N‐terminal Death Domains [26, 95]. Within these complexes, IRAK2 and IRAK3 are thought to recruit additional interactors and can serve as important conduits for connecting IRAK4 activation to the TRAF6 E3 ubiquitin ligase to enable downstream ubiquitylation to promote complex component turnover, relocalization and recruitment of effectors that contain ubiquitin recognition domains [67]. Plants have a particularly notable expansion of IRAK pseudokinases that may be inherent to their expanded reliance on innate over adaptive immunity [10]. Intriguingly, pseudokinase domains that appear in tandem with active kinase domains (tandem kinase‐pseudokinase proteins; TKPs) are also prevalent in plants, where they play a recurrent role in fungal pathogen resistance [96]. Although the molecular mechanisms of pseudokinases within TKPs are still being elucidated, early evidence suggests possible pseudokinase‐mediated recognition of fungal effector proteins [69]. Such examples reiterate the role of pseudokinases in nucleating key signaling complexes in many species and even across species boundaries.

### 4.3 Pseudokinases as Molecular Switches

While themselves catalytically‐inactive, pseudokinases are commonly modified by upstream regulators, including via phosphorylation by bona fide kinases, which can serve to modulate their functions. The best understood example is the pseudokinase, MLKL (mixed lineage kinase domain‐like), which serves as the terminal effector in the necroptosis cell death pathway [29, 97, 98, 99]. MLKL resides in cells in complex with its upstream regulator kinase, RIPK3, and upon exposure of the cell to stimuli, such as death ligands (e.g., TNF) or pathogen products (e.g., via Toll‐like receptor activation), MLKL is recruited to a high molecular weight platform termed the necrosome [79], where RIPK3 proceeds to phosphorylate MLKL in the activation loop of its pseudokinase domain (Figure 3C). This triggers a number of crucial conformational changes, including transition to a closed, active‐like conformation, release from RIPK3, assembly into a tetramer comprising a dimer of MLKL dimers and exposure of a lytic form of the N‐terminal four‐helix bundle domain [70, 71, 100]. This assembly is then trafficked to the plasma membrane [101, 102], where MLKL accumulates into large hotspots and the four‐helix bundle domains serve to permeabilize the plasma membrane [102], leading to cell death and expulsion of cellular contents to provoke an immune response. Whether similar transitions occur in other pseudokinases awaits detailed examination; for example, IRAK2 is known to be phosphorylated [66] but whether this modulates its interactions with other effectors within the Myddosome to dictate assembly of distinct signaling subcomplexes, or to modulate the activities of other enzymes within these complexes, remains to be established. Equally, proteins with analogous architecture to MLKL have been identified in plants and found to participate in host defense [103]. Whether a switching mechanism like that described for mammalian MLKL orthologs occurs amongst plant counterparts remains to be established. Furthermore, it is highly likely that some pseudokinases might exhibit a combination of allosteric, complex nucleation and conformational switching, although for illustrative purposes we have sought to designate separate functions here.

### 4.4 Pseudokinases as Inhibitors of Signaling Flux

Surprisingly few examples of pseudokinases functioning as suppressors of signaling by serving as decoys have been reported. Thus far, viral MLKL acting as an antagonist of cellular MLKL signaling stands as one of the few exemplars of how pseudokinases might interfere with cellular signaling. In this case, poxvirus‐encoded orthologs of MLKL (viral MLKL) bind to the upstream regulator of cellular MLKL, the RIPK3 kinase, and prevent engagement and activation of cellular MLKL by phosphorylation, thus blocking cell death [72, 104] (Figure 3D). Viral MLKL forms have evolved to serve as inhibitors of necroptotic signaling through loss of the killer four‐helix bundle domain and truncation of the activation loop compared to cellular MLKL, which eliminates the RIPK3 substrate sites that serve as a cue for MLKL to dissociate from RIPK3. Other pseudoenzymes offer illustrative examples of how holoenzyme activity can be thwarted by incorporation of a catalytically‐dead paralog instead of an active counterpart, as observed for the pseudo‐oxidoreductase, ALDH2*2, which is known to obstruct assembly of an active homotetramer of ALDH2*1 subunits [105]. However, no such examples are currently known for pseudokinases. Equally, we are not aware of examples of pseudokinases acting as substrate sinks to compromise signaling flux. In contrast, such a function has been proposed for pseudophosphatases [106], which can bind and prevent access of phosphosites to bona fide phosphatases. With ever‐increasing knowledge of pseudokinase function, especially driven by structural studies, we anticipate that examples of pseudokinases sequestering metabolites to prevent their downstream signaling functions will emerge. It will be intriguing to see whether high‐throughput computational methods for interrogating protein structure and interactions will reveal such roles for pseudokinases.

## 5 Pseudokinases as Emerging Targets for Therapeutic Intervention

The protein interaction functions of pseudokinases have opened our eyes to the likelihood that similar functions are performed by conventional protein kinases, but for many years have gone underappreciated because of the primary focus on their catalytic activities. Nonetheless, recent findings illustrate the importance of protein interactions to bona fide kinase functions, such as in scaffolding the assembly of signaling complexes as well as phosphorylation events regulating their conformations and related scaffolding functions. Consequently, there is great optimism that small molecules can serve as modulators of pseudokinase conformation and accordingly modulate downstream signaling outputs [107]. Even in cases where the pseudokinase domain ATP binding (“pseudoactive”) site is occluded by variant residues that preclude ATP binding, many examples have now emerged where pseudokinases are amenable to small molecule binding [23, 40]. Furthermore, because pseudokinase domain pseudoactive sites have not been functionally constrained to retain a conventional active site geometry required for catalytic activity, there is extensive variability in their geometries that could lend itself to highly specific small molecule modulators. These principles were leveraged in the development of the TYK2 pseudokinase domain‐targeting small molecule, Deucravacitinib, now FDA‐approved for the treatment of psoriasis and psoriatic arthritis [52]. This drug binds the TYK2 pseudokinase domain, but not those of the paralogs JAK1‐3, to lock the conformation and prevent activation of the adjacent tyrosine kinase domain, whose aberrant activity underlies inflammatory disease (Figure 3A). In addition to small molecule modulation of pseudokinase conformation and rigidity, including of STRAD*α*, KSR1/2, and the aforementioned TYK2 [52, 107, 108, 109], the divergence of pseudoactive sites is also highly attractive for developing specific chemical probes that can induce protein degradation. While conjugation to an E3 ubiquitin ligase recruiting moiety as a PROTAC can lead to pseudokinase turnover, even binding to small molecule inhibitors can be destabilizing and lead to protein degradation via the ubiquitin‐proteasome system, as evidenced for small molecule binders of TRIB2 [93, 110] and HER3 [111]. Additionally, targeted degradation of transmembrane receptor tyrosine pseudokinases has recently been explored as a therapeutic avenue for HER3, ROR1, and ROR2 with promising results [112, 113, 114]. While the inherent properties of pseudokinases preclude monitoring inhibition using simple phosphorylation‐based kinase assays in development pipelines, the increasing mechanistic understanding presented here, and advances in drug discovery technology, provide substantial promise for the development of pseudokinase‐targeted therapies.

## 6 Conclusion

Knowledge of pseudokinase signaling mechanisms has exploded dramatically since the CASK pseudokinase structure reported 18 years ago and WNK1 variant kinase structure 22 years ago. Because pseudokinases have evolved into distinct niches in signaling pathways, as well as within divergent organisms, much remains to be learned from mechanistic studies, especially structural characterization. Overexpression and genetic variants of many pseudokinases have led to their implication in dysregulated signaling and disease, raising the prospect that pseudokinases may be long‐underappreciated drug targets. The recent success of TYK2 inhibition has raised the prospect not only that modulating pseudokinase conformation is a viable strategy for drug development, but also that modulating the conformations of conventional protein kinases may similarly offer new avenues to therapeutic development.

## Funding

This work was supported by the National Health and Medical Research Council (2034104), Health Research Council of New Zealand (26‐0641), Marsden Fund (UOO2505), and National Institutes of Health (NIGMS R35GM139636, NCI U54CA274502).

## Conflicts of Interest

The authors declare no conflicts of interest.

## Data Availability

This review synthesizes data previously reported in the cited works and the associated structure coordinates deposited in the Protein Data Bank (PDB). No new data are reported in this article.
